# LasB activation in *Pseudomonas aeruginosa*: Quorum sensing-mediated release of an auto-activation inhibitor

**DOI:** 10.71150/jm.2411005

**Published:** 2025-02-27

**Authors:** Cheol Seung Lee, Xi-Hui Li, Chae-Ran Jeon, Joon-Hee Lee

**Affiliations:** 1 Department of Pharmacy, College of Pharmacy, Pusan National University, Busan 46241, Republic of Korea; 2 Research Institute for Drug Development, Pusan National University, Busan 46241, Republic of Korea

**Keywords:** *Pseudomonas aeruginosa*, elastase, LasB, protease, protease inhibition, quorum sensing

## Abstract

*Pseudomonas aeruginosa* secretes three major proteases: elastase B (LasB), protease IV (PIV), and elastase A (LasA), which play crucial roles in infection and pathogenesis. These proteases are activated sequentially from LasB in a proteolytic cascade, and LasB was previously thought to undergo auto-activation. However, our previous study suggested that LasB cannot auto-activate independently but requires additional quorum sensing (QS)-dependent factors for activation, as LasB remained inactive in QS-deficient *P. aeruginosa* (QS^-^) even under artificial overexpression. In this study, we provide evidence for the existence of a LasB inhibitor in QS^-^ mutants: inactive LasB overexpressed in QS^-^ strains was in its processed form and could be reactivated upon purification; when full-length LasB was overexpressed in *Escherichia coli*, a heterologous bacterium lacking both LasB activators and inhibitors, the protein underwent normal processing and activation; and purified active LasB was significantly inhibited by culture supernatant (CS) from QS^-^ strains but not by CS from QS^+^ strains. These findings demonstrate that a LasB inhibitor exists in QS^-^ strains, and in its absence, LasB can undergo auto-activation without requiring an activator. Based on these results, we propose an updated hypothesis: the QS-dependent LasB activator functions by removing the LasB inhibitor rather than acting directly on LasB itself, thus preventing premature LasB activation until QS response is initiated.

## Introduction

*Pseudomonas aeruginosa* is a notorious Gram-negative pathogen that causes serious infections in people with burn wounds, cystic fibrosis, immunodeficiency, and chronic pulmonary disorders (Qin et al., 2022), and known to have multiple antibiotic resistance mechanisms, produce numerous virulence factors, and form robust biofilm, a very protective life mode (Horcajada et al., 2019). Among the virulence factors produced by *P. aeruginosa*, three extracellular proteases, elastase B (LasB), lysine-specific endopeptidase (PIV) and staphylolysin (LasA) are crucially implicated in the pathogenicity by damaging host tissues and immune components (Kessler et al., 1998; Li and Lee, 2019).

The expression and activation of these 3 proteases are elaborately regulated by multiple complex mechanisms. First, the transcription of these protease genes is strictly controlled by quorum sensing (QS) that is a cell-to-cell communication mechanism to regulate gene expression in a cell density-dependent manner (Miller and Bassler, 2001; Moradali et al., 2017). In *P. aeruginosa*, two principal QS signal synthases, LasI and RhlI (encoded by *lasI* and *rhI*, respectively) continuously synthesize *N*-3-oxododecanoyl homoserine lactone and N-butyryl-homoserine lactone, respectively as the QS signals (Miranda et al., 2022; Williams et al., 2004). When these QS signals reach a critical concentration, they enter cells to bind to their cognate receptors and activate the transcription of many QS regulon genes, including *lasB* (encoding LasB), *lasA* (encoding LasA), and *piv* (encoding PIV) (Albus et al., 1997; Ding et al., 2018; Kostylev et al., 2019).

The second regulation occurs at extracellular space during secretion. Since PIV, LasB, and LasA all have a common domain organization (a signal peptide (SP) at the N-terminus, a propeptide (PP) domain in the middle, and a mature protease domain at the C-terminus), they are initially expressed in a full length containing all domains (prepro-forms) that is inactive. But during secretion, these prepro-forms are processed by proteolytic cleavage to produce mature active forms. For example, mature LasB is a 33 kDa zinc-metalloprotease but LasB is initially synthesized as a prepro-form of 53.6 kDa. The 2.4 kDa SP is eliminated upon passage through the inner membrane into the periplasm to make pro-LasB, and the 18 kDa PP is subsequently cleaved off by proteolysis in periplasm to make mature LasB (Kessler et al., 1998; McIver et al., 1995). Before cleavage, the PP plays an essential role for the proper folding of LasB in the periplasm like some proteases mentioned above, and this proper folding allows for further autoproteolytic processing of the pro-LasB to mature LasB (McIver et al., 1991, 1995). Therefore, the cleaved PP functions as an inhibitor for LasB and intramolecular chaperone for LasB activity at the same time (Kessler and Safrin, 1988; McIver et al., 1991, 1995). PIV and LasA undergo similar processes: They are activated through propeptide cleavage during secretion in a sequential proteolytic cascade initiated by LasB. In this cascade, LasB activates pro-PIV to form active PIV, and subsequently, both LasB and PIV contribute to the activation of pro-LasA to form active LasA (Li and Lee, 2019).

So far, LasB itself has been suggested to be auto-activated and considered an initial triggering activator in this cascade activation (McIver et al., 1991, 1993). In this LasB activation model, LasB was considered to be the topmost factor that initiates activation cascade first, and was thought to not require other factors for its activation. However, our previous study has demonstrated that LasB is not activated in the QS-deficient strain of *P. aeruginosa* (QS^-^), even with artificial overexpression (Li and Lee, 2019). Interestingly, this inactive LasB in QS^-^ strain was fully activated by the addition of the culture supernatant of QS^+^ strain (Li and Lee, 2019), suggesting the existence of QS-dependent activator. Therefore, the current model, which assumes LasB auto-activation, needed to be revised. In this study, we re-examined the activation mechanism of LasB and found that the extracellular activation of LasB involves not only a QS-dependent activator but also a QS-independent inhibitor. This indicates that LasB is not the primary initiator in the regulatory mechanism, but rather there exists an additional upstream regulatory system consisting of both inhibitory and activating factors. Based on these findings, we propose a new model for the initial activation mechanism of LasB.

## Materials and Methods

### Bacterial strains, plasmids and culture conditions

Bacterial strains and plasmids utilized in this study are listed in Table 1. Throughout this study, PAO1 or Δ*lasB* mutant was used for the QS^+^ strain, and MW1 (*lasI*^-^, *rhlI*^-^) was used for the QS^-^ strain. Bacterial cells in general were cultured with vigorous shaking at 37°C under 180 rpm. Luria-Bertani (LB) medium (0.5% yeast extract, 0.5% NaCl, and bacto-tryptone 1%) was used to grow the bacteria.1.5% (w/v) agar was added to solidify LB media. The bacterial growth was measured by optical density at 600 nm (OD_600_) using a spectrophotometer (OPTIZEN). Gentamicin and tetracycline were added at 10 µg/ml for *E. coli* and 50 µg/ml for *P. aeruginosa*, respectively, and ampicillin, 100 µg/ml, and carbenicillin, 150 µg/ml. In order to induce protein expression, 0.5 mM Isopropyl-1-thio- β-D-galactopyranoside (IPTG) was added at OD_600_ = 0.5.

### Culture supernatant (CS) preparation

Bacterial cells were cultivated in 5 ml of LB with vigorous shaking at 37ºC overnight and diluted at 1:100 ratio into 5 ml fresh LB broth with 0.4% arabinose for the main culture. After further overnight culture with vigorous shaking at 37ºC, cells were removed by centrifugation at 13,000 rpm for 2 min at 4ºC and the CSs were filtered through 0.22 μm syringe filter (Satorious) and 10 times concentrated by 10 kDa cut-off centricon (Vivaspin^®^, Satorious).

### Overexpression and purification of LasB in BL21

LasB were overexpressed with the C-terminal histidine tag from the plasmid pQF21c-LasB (Table 1). The pQF21c-LasB plasmid was introduced to *E. coli* BL21 by transformation. The transformant was inoculated into 100 ml of LB with 100 μg/ml ampicillin (150 μg/ml carbenicillin) and cultured at 37ºC with vigorous shaking up to OD_600_ = 0.5. Then, IPTG was added at 0.5 mM to induce the protein expression and the cells were further cultivated at 37ºC with shaking. After 16-h cultivation, cells were harvested by centrifugation at 14,000 rpm for 20 min at 4ºC and washed with 20 mM Tris-HCl (pH 8.0). The cells were resuspended in a binding buffer (20 mM Tris-HCl, 5% glycerol, pH 8.0) and disrupted by sonication. The soluble fraction was separated from insoluble fraction by centrifugation at 14,000 rpm for 20 min at 4ºC. The soluble fraction was loaded on a Ni-NTA column (Invitrogen) and washed with low concentration of imidazole (20 mM Tris-HCl, 20 mM imidazole, 5% glycerol, pH 8.0). The tightly bound proteins were eluted with increasing concentrations of imidazole (20 mM Tris-HCl, 50–500 mM imidazole, 5% glycerol, pH 8.0). The fractions containing pure LasB were collected, dialyzed in storage buffer (20 mM Tris-HCl, 5% glycerol, pH 8.0) and stored at -80ºC.

### Overexpression and purification of LasB in *P. aeruginosa*

For the LasB purification, LasB-overexpressing plasmid (pSP201, Table 1) was introduced into the PAO1 or MW1, and the transformed cells were cultivated in 300 ml of LB medium at 37°C with vigorous shaking under 180 rpm. Arabinose was added at 0.8% to induce LasB for 16 h. Cells were then removed by centrifugation and the culture supernatant was taken. LasB was precipitated by slow addition of ammonium sulfate ((NH_4_)_2_SO_2_) and pelleted by centrifugation. The pellet was dissolved in 20 mM Tris-HCl (pH 8.0) and the remained salt was completely removed by dialysis. This LasB-containing solution was applied to DEAE sepharose column chromatography and eluted by salt gradient (0–1 M NaCl) by using FPLC (Fast Protein Liquid Chromatography). The fractions containing pure LasB were collected and dialyzed in the storage buffer (20 mM Tris-HCl and 5% glycerol, pH 8.0). The purified LasB was aliquoted and stored at -80°C.

### LasB activity assay

The elastolytic activity of LasB was measured using the Elastin-Congo red method, as previously described (Li and Lee, 2019; Shin et al., 2022). The CSs prepared from the indicated strains or purified proteins were incubated with 10 mg/ml elastin-Congo red (EPC, USA) at 37ºC for 16 h in 0.3 ml of 10 mM Tris-HCl (pH 8.0). Any precipitation was removed by centrifugation at 14,000 × g for 10 min and absorbance at 490 nm (A_490_) was measured using an iMarkTM microplate reader (Bio-Rad). LasB activity was calculated by following equation: LasB activity (%) = [(A_495_ of the elastolytic reaction with sample - A_495_ of the elastolytic reaction with LB) / (A_495_ of elastolytic reaction with PAO1 - A_495_ of the elastolytic reaction with LB)] × 100. HCl, 5% glycerol, pH 8.0) and stored at -80ºC.

### Statistical analysis

The significance of difference was determined by using student’s *t*-test (two sample assuming equal variances) in MS Office Excel (Microsoft). When *p*-values < 0.05, it was considered significant.

## Results

### The LasB inhibitor is present in QS-deficient strains, where LasB is not auto-activated even after auto-processing

**Fig. 1.**
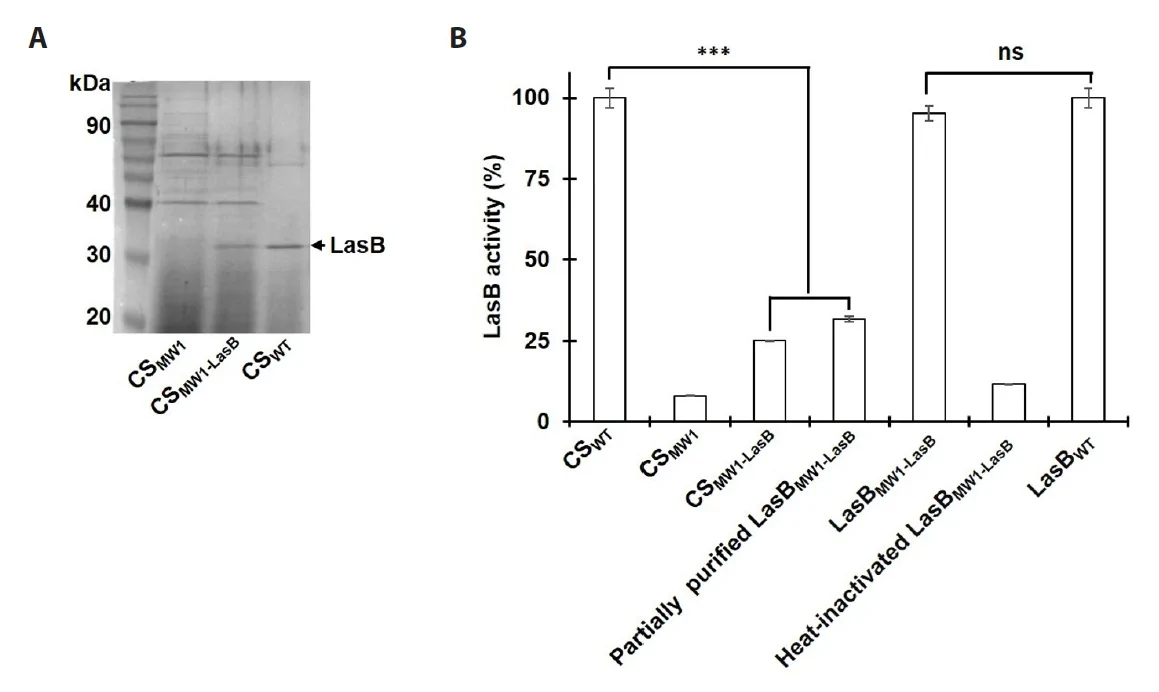
LasB is processed but remains inactive in the QS-deficient strain, and becomes activated upon purification. The CSs were prepared from PAO1 (CS_WT_), MW1 (CS_MW1_), MW1 harboring a LasB-overexprsessing plasmid, pSP101 (CS_MW1-LasB_), and LasB was purified from CS_MW1-LasB_ and CS_WT_ through (NH_4_)_2_SO_4_ precipitation and Ni-NTA column purification. (A) LasB in each CS was observed in 15% SDS-PAGE before purification. (B) LasB activity was measured and compared among the CSs, LasB purified from CS_MW1-LasB_ (LasB_MW1-LasB_), LasB purified from CS_WT_ (LasB_WT_), and partially purified LasB_MW1-LasB_ obtained from (NH_4_)_2_SO_4_ precipitation. CSs containing 2 µg of total protein and 100 ng of purified protein were used. Heat inactivation was done at 100°C for 10 min. ^***^, *P* < 0.005; ns, no significance.

Our previous study demonstrated that LasB remains inactive in the QS-deficient strain of *P. aeruginosa* (QS^-^; MW1, Table 1), even under artificial overexpression conditions (Li and Lee, 2019). However, the addition of culture supernatant (CS) from the QS^+^ strain fully restored LasB activity in the QS^-^ strain (Li and Lee, 2019), suggesting the existence of QS-dependent activator. Interestingly, examination of LasB status in the CS of the LasB-overexpressing QS^-^ strain (CS_MW1-LasB_) revealed that LasB existed in its mature form, with both SP and PP already cleaved (Fig. 1A). This finding demonstrated that despite proper processing, LasB remained in an inactive state, strongly suggesting that LasB activation requires mechanisms beyond mere proteolytic processing of pro-LasB. Therefore, we realized that we needed to distinguish between auto-processing, which refers to self-cleavage of PP, and auto-activation, which refers to self-activation.

More intriguingly, the inactive LasB regained its activity upon purification from the QS^-^ strain. As demonstrated in Fig. 1B, LasB purified from CS_MW1-LasB_ exhibited minimal activity during intermediate purification steps; however, once fully purified, it displayed activity comparable to that of LasB purified from the CS of the LasB-overexpressing wild-type (CS_WT-LasB_). These findings suggested that LasB was under suppression by an inhibitory factor unique to the QS^-^ strain, and that removal of this factor through purification was sufficient for LasB activation without additional activating factors. Based on these observations, we hypothesized that in QS^+^ strains, QS-dependent expression of LasB-activating factors eliminates the inhibitory factor, enabling immediate activation of LasB following processing. Conversely, in QS^-^ strains, the persistent presence of the inhibitory factor maintains LasB in an inactive state even after proper processing.

### LasB produced in the heterologous host *E. coli*, which lacks inhibitor, was auto-processed and auto-activated

**Fig. 2.**
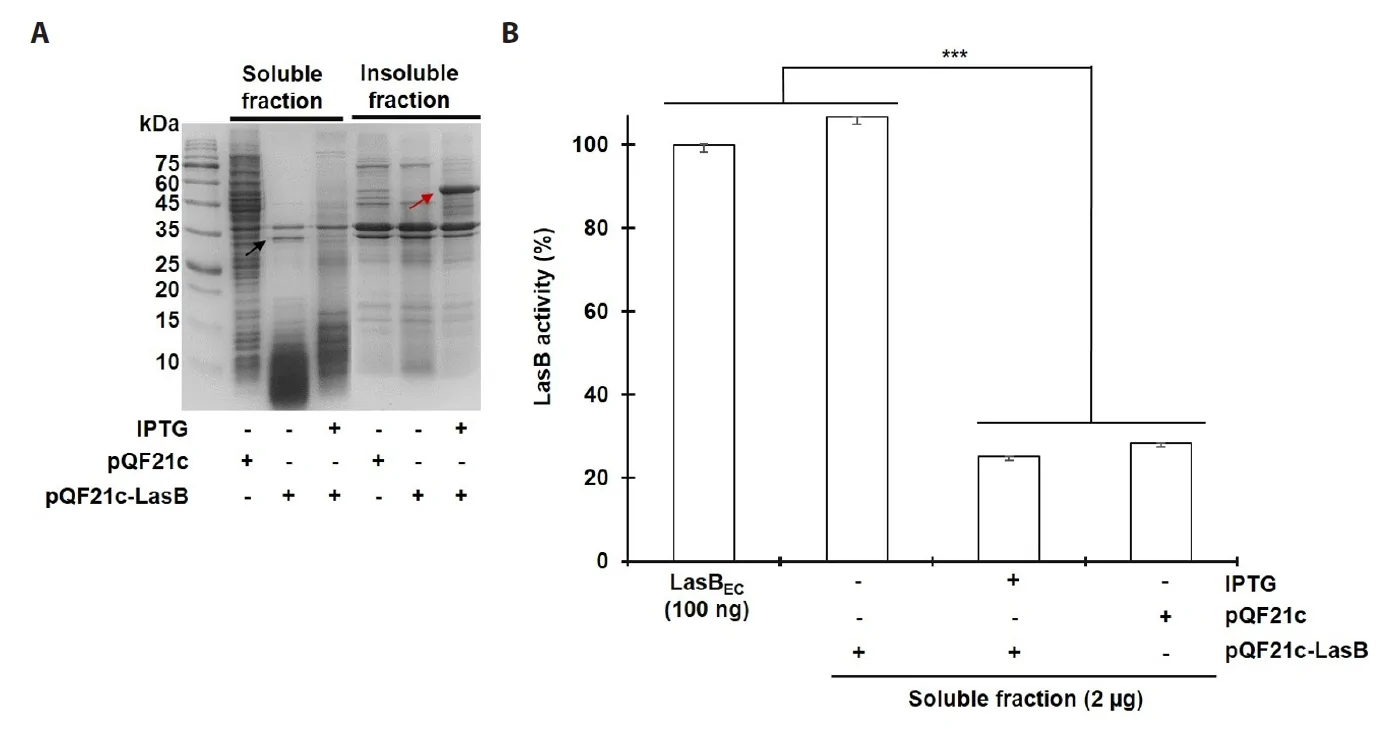
When overexpressed in *E. coli*, LasB undergoes both processing and activation. LasB was overexpressed by using pQF21c-LasB in *E. coli* BL21. (A) crude extract was separated into soluble and insoluble fractions and subjected to 15% SDS-PAGE. Mature LasB and unprocessed prepro-LasB are indicated by black and red arrows, respectively. *E. coli* BL21 transformed with pQF21c as a vector control and was analyzed in parallel. (B) LasB activity was measured in the soluble fraction and LasB_WT_ that is LasB purified from the uninduced soluble fractions. ^***^, *P* < 0.005.

To verify the existence of LasB inhibitor, LasB was overexpressed in *E. coli* rather than *P. aeruginosa*. Since *E. coli* is a heterologous host presumed to lack LasB inhibitor, the LasB expressed in *E. coli* was expected to maintain its activity once properly processed. When we overexpressed LasB in *E. coli*, and separate the whole cytoplasmic proteins of the LasB-overexpressing *E. coli* cells into soluble and insoluble fractions, LasB highly overexpressed by IPTG addition, existed mainly as unprocessed prepro-form (53 kDa) in the insoluble fraction, whereas without IPTG induction, the slightly overexpressed LasB was predominantly found as the processed mature form (33 kDa) in the soluble fraction (Fig. 2A). When measuring LasB activity in the soluble fraction, the slightly overexpressed LasB without IPTG showed high levels of activity, whereas the highly overexpressed LasB with IPTG exhibited very low activity (Fig. 2B). All insoluble fractions showed no LasB activity (data not shown). When the mature form of LasB was purified from the soluble fraction, it also showed high activity (Fig. 2B). This result clearly demonstrates that in *E. coli*, which lacks *P. aeruginosa* factors including inhibitors, LasB undergoes auto-processing and the auto-processed form retain activity.

**Fig. 3.**
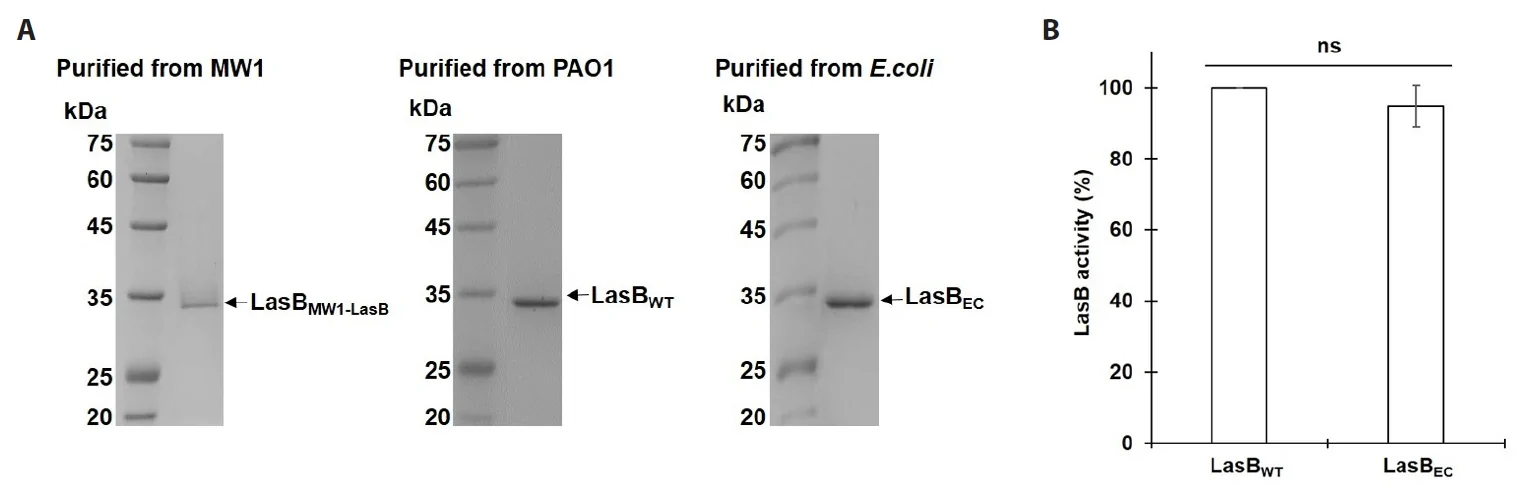
The size and activity of LasB were identical whether purified from *E. coli* or *P. aeruginosa*. (A) SDS-PAGE analysis were performed to compare LasB proteins purified from different sources: *P. aeruginosa* MW1 (LasB_MW1-LasB_, see Fig. 1), *P. aeruginosa* PAO1 (LasB_WT_, see Fig. 1), or *E. coli* BL21 (LasB_EC_, see Fig. 2). (B) the LasB activity was measured and compared using 100 ng of purified LasB_WT_ and LasB_EC_. For comparison with LasB_MW1-LasB_, refer to Fig. 1B. ns, no significance.

The LasB that was expressed and processed in *E. coli* showed no differences in size compared to the LasB processed in wild type *P. aeruginosa* or the QS^-^ strain (Fig. 3A). Furthermore, LasB processed in *E. coli* and wild type *P. aeruginosa* showed no differences in activity, and its activity was also identical to that of LasB activated after purification from the QS^-^ strain (Fig. 3B & 1B). Therefore, despite all conditions being identical, the fact that LasB expressed and processed in the QS^-^ strain shows low activity before purification clearly indicates the presence of an inhibitor in the QS^-^ strain.

### LasB is suppressed by a QS-independent inhibitor and becomes activated when the inhibitor is removed by a QS-dependent activator

**Fig. 4.**
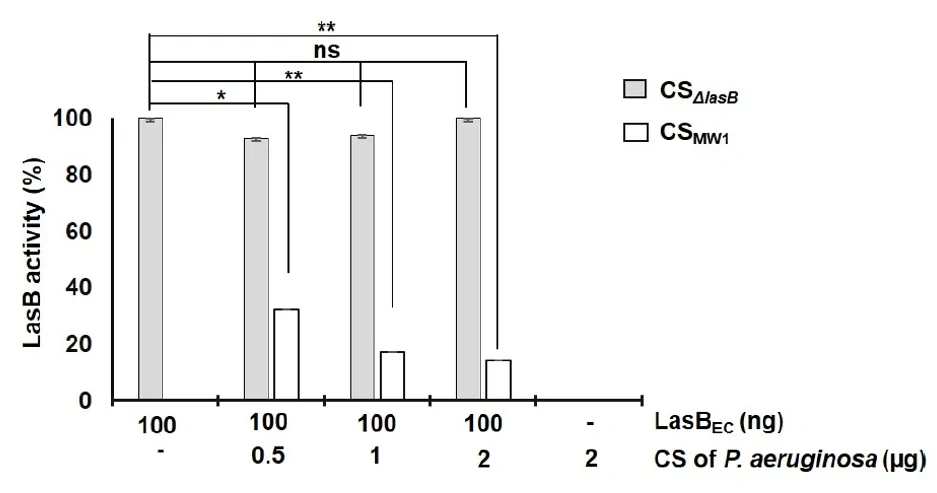
The LasB inhibitor exists only in the QS^-^ strain and not in the QS^+^ strain. The purified LasB from *E. coli* (LasB_EC_) was mixed with CS prepared from Δ*lasB* mutant (CS_Δ*lasB*_) or CS prepared from MW1 (CS_MW1_), incubated for 30 min, and measured for LasB activity. The amount of CS was presented as the total amount of protein contained in CS. ^*^, *P* < 0.05; ^**^, *P* < 0.01; ns, no significance.

In our previous study, we demonstrated the existence of a QS-dependent LasB activator (Li and Lee, 2019). In this study, we additionally discovered the presence of a LasB inhibitor that exists only in QS^-^ strains. Therefore, we investigated how these two factors are involved in the activation of LasB. We hypothesized that while *P. aeruginosa* produces a QS-independent inhibitor to suppress LasB, the QS-dependent activator removes this inhibitor, thereby releasing LasB from inhibition. In order to prove this hypothesis, we treated purified active LasB with CS prepared from the QS^-^ strain (CS_MW1_). As a control, we similarly treated CS from the Δ*lasB* mutant strain (CS_Δ*lasB*_). Δ*lasB* mutant is a QS^+^ strain, but lacks LasB activity. As shown in Fig. 4, only CS_MW1_ could inhibit LasB activity in a dose-dependent manner, whereas there was no inhibition with the CS_Δ*lasB*_ treatment. These results clearly demonstrate that the LasB inhibitor exists only in the QS^-^ strain and not in the QS^+^ strain.

**Fig. 5.**
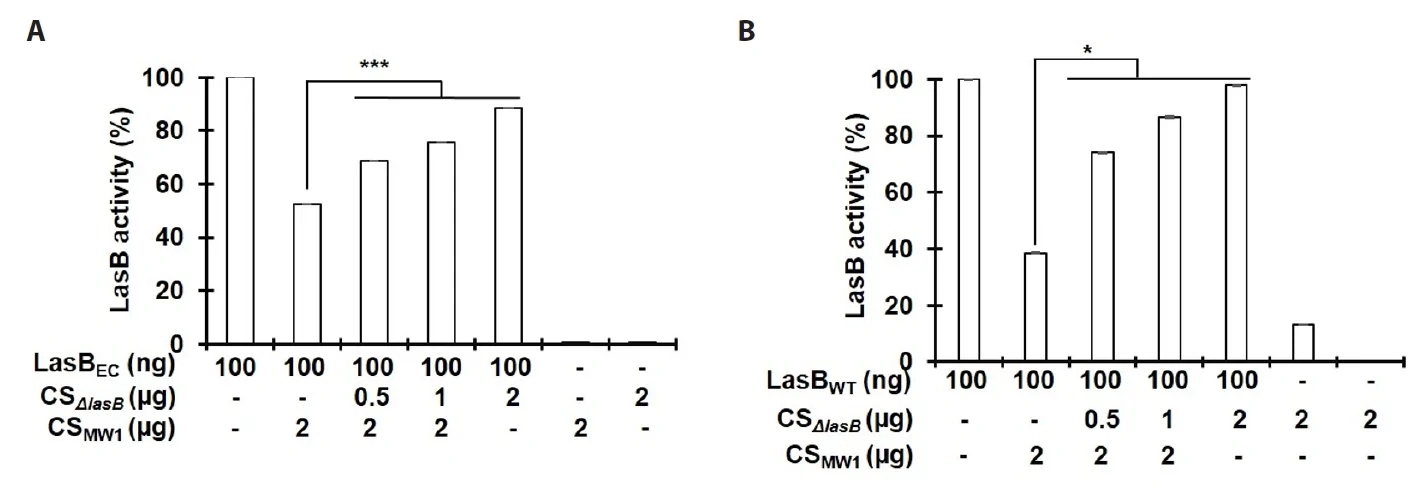
Elimination of LasB inhibitor by CS of QS^+^ strain. CS_MW1_ was incubated for 30 min with increasing amount of CS_Δ*lasB*_ and added to 100 ng of LasB_EC_ (A) or LasB_WT_ (B). After 4-h incubation, LasB activity was measured. The amount of CS was presented as the total amount of protein contained in CS. ^*^, *P* < 0.05; ^***^, *P* < 0.005.

In our previous study, we presented evidence for the existence of a QS-dependent LasB activator (Li and Lee, 2019). This factor present in QS^+^ strain could activate the inactive LasB present in LasB-overexpressing MW1 (Li and Lee, 2019). Nonetheless, the above results in this study suggest that LasB undergoes normal auto-processing and auto-activation in the absence of inhibitors, without requiring an activator. An explanation for both of these seemingly contradictory statements is to assume that the LasB activator serves to remove the inhibitor rather than affecting LasB itself. To prove this hypothesis, the CS_MW1_ that contain the LasB inhibitor was incubated with CS_Δ*lasB*_ that contains the LasB activator. By doing this, it was expected that the LasB inhibitor would be eliminated by the LasB activator. After incubation, when the two purified active LasBs, LasB_EC_, and LasB_WT_ were treated with the mixture of CS_MW1_ and CS_Δ*lasB*_, the inhibition on LasB activity by CS_MW1_ was gradually decreased according to the increasing amount of CS_Δ*lasB*_ (Fig. 5). This result clearly demonstrates that the inhibitor in the QS^-^ strain is removed by the QS-dependent activator. Collectively, our findings suggest that the LasB activator is a QS-dependent factor that removes the LasB inhibitor, rather than directly processing LasB itself. Furthermore, our data indicate that the activator functions after LasB processing has occurred, since LasB processing occurred normally even in the QS^-^ strain.

## Discussion

**Fig. 6.**
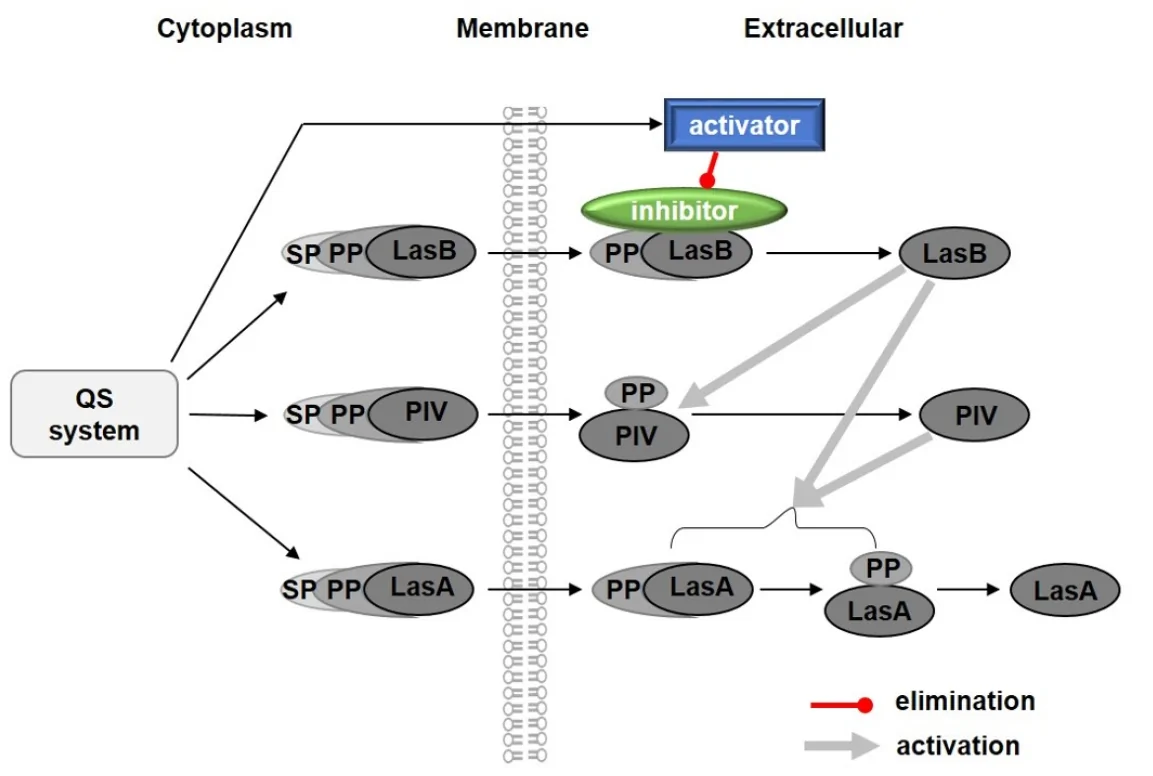
Updated model for the extracellular activation of LasB in *P. aeruginosa*. The previously proposed model is shown in black and white (Li & Lee, 2019), while the content added by this study is shown in color. The key addition is that in the extracellular space, LasB activity is inhibited by a QS-independent inhibitor, but when a QS-dependent activator is expressed, it removes the inhibitor, thereby activating LasB.

*P. aeruginosa* is an opportunistic pathogen, and LasB serves as one of its crucial virulence factors. The expression of LasB is regulated by the quorum sensing (QS) system in *P. aeruginosa* (Yang et al., 2021). Although it has been suggested that the full length prepro-LasB is auto-processed and auto-activated (McIver et al., 1991), our previous study has demonstrated that LasB is inactive when expressed in the QS^-^ mutant, and suggested that LasB cannot auto-activate alone and requires a QS-dependent factor for activation (Li and Lee, 2019). Based on this previous study, we proposed a new model for the QS-dependent extracellular activation of major proteases, LasB, PIV, and LasA, as follows (Fig. 6): In the cytoplasm, the prepro-forms of the proteases are expressed under transcriptional control of QS, and their SPs are removed during translocation over the cytoplasmic membrane. In the extracellular space, the pro-form of LasB is first activated by unknown QS-dependent activating factors. The activated LasB, in turn, activates pro-PIV to PIV, and the activated PIV activates pro-LasA into active LasA. LasB also plays an auxiliary role in activation of LasA.

However, our model suggesting the existence of QS-dependent activating factors conflicted with the previously established model of LasB auto-processing and auto-activation (McIver et al., 1991, 1993). To address this inconsistency, we verified the presence of an inhibitor in QS^-^ strain and subsequently proposed an updated model that incorporates the cooperative involvement of both inhibitor and activator (Fig. 6). A crucial aspect of this updated model is the distinction between LasB auto-processing and auto-activation. Although LasB can undergo auto-processing independently without other factors, our model demonstrates that even after processing, LasB remains in an inactive state in QS^-^ strains due to the presence of an inhibitor. In contrast to this, in the QS^+^ wild type, LasB becomes activated because the inhibitor is quickly removed by the QS-dependent activator (Fig. 6).

A key evidence for this updated model is that the inactive LasB overexpressed in QS^-^ strain was already in its processed form and reactivated when purified (Fig. 1A & 1B). Another key evidence is that when full length LasB was overexpressed in *E. coli*, a heterologous bacterium that has neither activator nor inhibitor for LasB, LasB was normally processed and activated, as proposed in the previous model (Fig. 2A & 2B). These evidences clearly establish the existence of a LasB inhibitor, even though it may not be readily observable in QS^+^ wild type strains. This is because the inhibitor is rapidly eliminated by activators expressed in QS^+^ strains. In this study, we confirmed this mechanism by utilizing QS^-^ strains, thereby providing an updated and more precise understanding of the LasB activation mechanism.

## Figures and Tables

**Table 1.** Bacterial strains and plasmids used in this study

Name	Description	References
***Pseudomonas aeruginosa***		
PAO1	Wild type* P. aeruginosa*, QS^+^	(Pearson and Pesci et al., 1997)
MW1	*lasI^-^, rhlI^-^* double mutant of PAO1, Tc^R^, QS^-^	(Whiteley et al., 1999)
*ΔlasB*	*lasB^-^* mutant of PAO1, Tc^R^, QS^+^	(Li and Lee, 2019)
***Escherichia coli***		
DH5α	*supE44*Δ*lacU*169(80*lacZ*ΔM15) *hsdR17 recA1* *gyrA96thi-1 relA1*	Lab. collection
BL21(DE3)	F^-^*omp*T* hsd*S_B_ (r_B_^-^ m_B_^-^) *dcm* *gal* λ (DE3)	Lab. collection
**Plasmids**		
pJN105	*araC*-pBAD promoter fusion plasmid, Gm^R^	(Newman and Fuqua, 1999)
pSP201	PA3724 (*lasB*) in pJN105, Gm^R^	(Park et al., 2014)
pQF21c	Overexpression plasmid for C-terminal histidine-tagged protein (a modified pET21c with a broad-host-range replication origin, Ori_1600_), replicable in *P. aeruginosa*, Cb^R^ (Ap^R^ for *E. coli*)	(Park et al., 2014)
pQF21c-LasB	*lasB* in pQF21c, Cb^R^ (Ap^R^ for *E. coli*)	(Li and Lee, 2019)

Tc, Tetracycline; Gm, Gentamicin; Cb, Carbenicillin; Ap, Ampicillin; Km, Kanamycin
